# NFAT-mediated defects in erythropoiesis cause anemia in *Il2*^−/−^ mice

**DOI:** 10.18632/oncotarget.23745

**Published:** 2017-12-28

**Authors:** Sabrina Giampaolo, Gabriela Wójcik, Stefan Klein-Hessling, Edgar Serfling, Amiya K. Patra

**Affiliations:** ^1^ Department of Molecular Pathology, Institute of Pathology, University of Würzburg, 97080 Würzburg, Germany; ^2^ Institute of Translational and Stratified Medicine, Peninsula Schools of Medicine and Dentistry, University of Plymouth, Plymouth PL6 8BU, UK; ^3^ Comprehensive Cancer Center Mainfranken, University of Würzburg, 97080 Würzburg, Germany

**Keywords:** erythropoiesis, anemia, IL-2, integrin, cAMP

## Abstract

The role of NFAT family transcription factors in erythropoiesis is so far unknown, although their involvement has been suggested previously. We have shown recently that *Il2*^-/-^ mice develop severe anemia due to defects in KLF1 activity during BM erythropoiesis. Although, KLF1 activity is indispensable for erythropoiesis, the molecular details of *Klf1* expression have not yet been elucidated. Here we show that an enhanced NFATc1 activity induced by increased integrin-cAMP signaling plays a critical role in the dysregulation of *Klf1* expression and thereby cause anemia in *Il2*^-/-^ mice. Interestingly, enhanced NFATc1 activity augmented apoptosis of immature erythrocytes in *Il2*^-/-^ mice. On the other hand, ablation of NFATc1 activity enhanced differentiation of Ter119^+^ cells in BM. Restoring IL-2 signaling in *Il2*^-/-^ mice reversed the increase in cAMP-NFAT signaling and facilitated normal erythropoiesis. Altogether, our study identified an NFAT-mediated negative signaling axis, manipulation of which could facilitate erythropoiesis and prevent anemia development.

## INTRODUCTION

Erythropoiesis in bone marrow (BM) is regulated by a host of signaling pathways [1–4], and key erythroid lineage-specific transcription factors (TF) such as the erythroid Krüppel-like factor (EKLF/KLF1), GATA1, RUNX1, FOG1, SCL and NF-E2 etc., [1, 2, 5–7]. Additionally, growth factors such as erythropoietin (Epo) play an indispensable role in facilitating differentiation of immature erythrocytes to become mature red blood cells (RBC) [8]. Genetic ablation or inactivating mutations in any of these molecules have been reported to be lethal for the survival of animals as these abnormalities result in severely defective erythropoiesis [9–15].

IL-2 signaling plays a critical role in the survival, proliferation and function of a variety of immune cells [16–18]. Also, it is a critical factor in maintaining immune homeostasis because of its influence on regulatory T (T_reg_) cells [19]. *Il2*^−/−^ and all other mice with defective IL-2 signaling develop severe autoimmune pathologies due to lack of optimal T_reg_ cell activity [20–23]. Severe immune pathologies have also been reported in case of humans having defects in IL-2 signaling [24]. We have recently shown that mice lacking IL-2 signaling have strong defects in erythrocyte differentiation and they develop anemia very early in life [25]. Though, the reasons behind this are not completely known, we have reported that *Klf1* suppression is a major factor for anemia development in *Il2*^−/−^ mice [25]. However, in addition to dysregulated KLF1 activity, whether other signaling molecules are also involved is so far unknown. Further, the molecular mechanism involved in *Klf1* suppression in IL-2-deficient erythroid precursor cells has also not been elucidated.

Although, many TFs are involved in the regulation of BM erythropoiesis, the contributions of nuclear factor of activated T cell (NFAT) family TFs (NFATc1, NFATc2, NFATc3, NFATc4 and NFAT5) has so far not been investigated. Besides NFAT5, all other NFAT proteins are regulated by a calcium (Ca^2+^)-calcineurin-mediated mechanism [26, 27]. In mature lymphocytes, immune receptor (T cell receptor; TCR or B cell receptors; BCR) ligation with cognate ligands results in an increase in intracellular calcium levels in a phospholipase C-γ (PLC-γ)-dependent manner, which subsequently activate the serine-threonine phosphatase calcineurin. Active calcineurin dephosphorylates multiple serine residues in the cytoplasmic NFAT proteins facilitating their activation and nuclear translocation. Nuclear NFAT regulates gene expression related to cytokine production, cell cycle, cell death, and cell differentiation etc, [28, 29]. Besides the calcineurin-mediated pathway, NFAT proteins can also be activated by cytokines as has been reported in the context of preTCR-negative thymocytes [30]. On the other hand, NFAT5 mostly expressed in non-hematopoietic lineage cells is activated upon osmotic stress [31]. Alterations in NFAT activity have been reported to induce pathological conditions ranging from immunodeficiency to cancer [30, 32–34]. However, despite extensive analysis of the role of NFAT proteins in lymphocyte development and function, their role in erythropoiesis still need to be investigated. Here, by analyzing *Il2*^−/−^ mice we show that an enhanced integrin-cAMP-NFAT signaling axis not only downregulated *Klf1* expression but also increased the apoptosis of *Il2*^−/−^ erythrocyte precursors. Our findings suggest that NFAT hyperactivity is a key mechanism that blocks erythrocyte differentiation and promotes anemia development in *Il2*^−/−^ mice, which might also be operative in inducing anemia in humans.

## RESULTS

### Impaired NFAT activity in *Il2*^-/-^ immature erythrocytes

**Figure 1:**
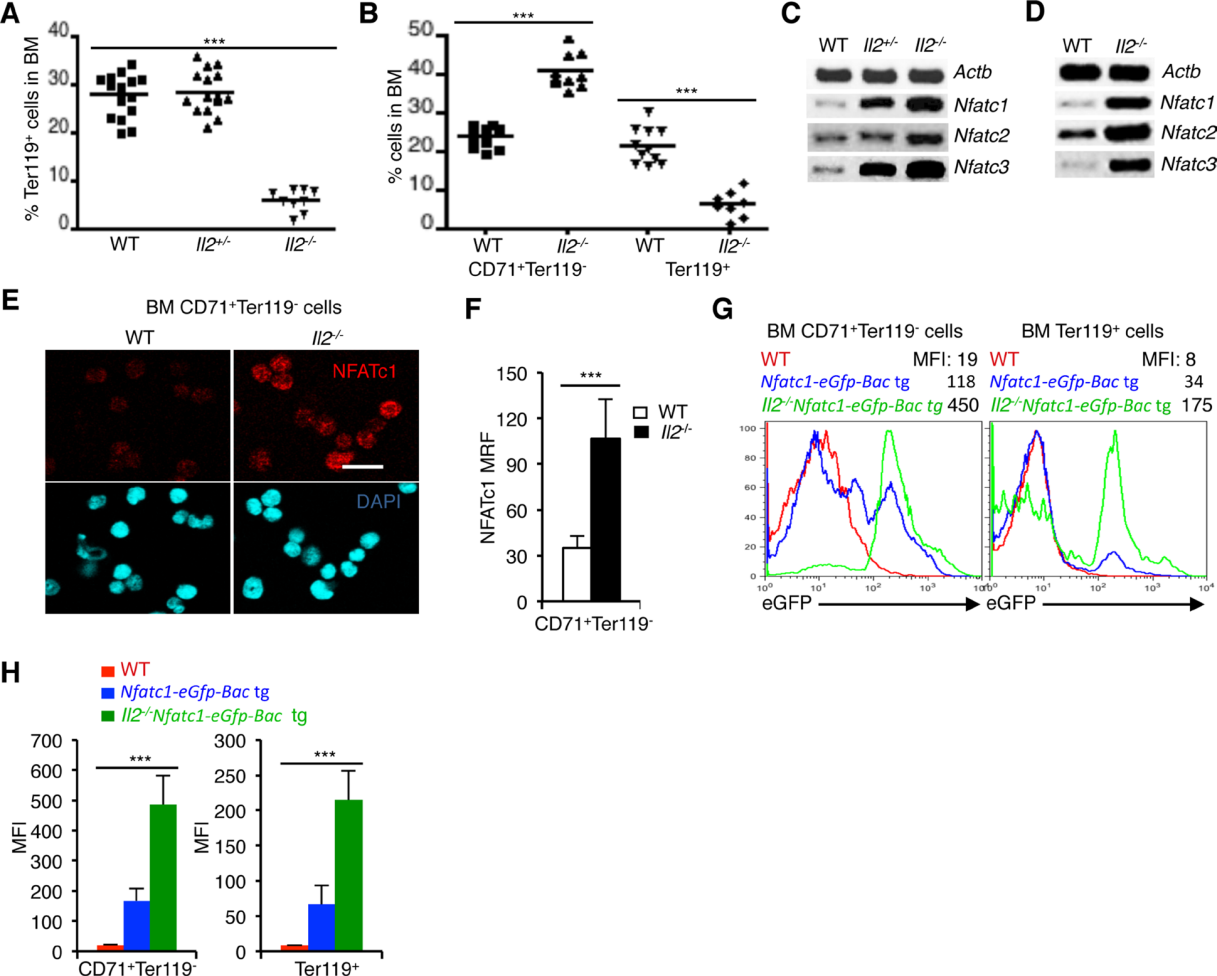
NFAT expression in erythrocytes (**A**) Distribution of Ter119^+^ cells in the BM of *Il2*^−/−^ mice compared to WT and *Il2*^+/-^ littermate controls. (**B**) Distribution of CD71^+^Ter119^-^ and Ter119^+^ (CD71^+^Ter119^+^ + CD71^-^Ter119^+^) erythrocyte precursor cells in the BM of WT and *Il2*^−/−^ mice. (**C**) Expression levels of *Nfatc1*, *Nfatc2* and *Nfatc3* mRNA in total BM cells from indicated mice. (**D**) *Nfatc1*, *Nfatc2* and *Nfatc3* gene expression in isolated BM Ter119^+^ cells from WT and *Il2*^−/−^ mice. (**E**) Immunofluorescence analysis of NFATc1 levels in sorted BM CD71^+^Ter119^-^ cells from WT and *Il2*^−/−^ mice. DAPI fluorescence indicates nuclear staining. Scale bar, 10 μm. (**F**) Quantification of NFATc1 mean relative fluorescence (MRF) in BM CD71^+^Ter119^-^ cells from WT (*n* = 52) and *Il2*^−/−^ (*n* = 42) mice. (**G**) GFP expression levels in BM CD71^+^Ter119^-^ and Ter119^+^ (CD71^+^Ter119^+^ + CD71^-^Ter119^+^) cells from *Il2*^−/−^*Nfatc1-eGfp-Bac* tg reporter mice compared to WT and *Nfatc1-eGfp-Bac* tg mice. (**H**) Quantification of GFP expression levels in BM CD71^+^Ter119^-^ and Ter119^+^ (CD71^+^Ter119^+^ + CD71^-^Ter119^+^) cells from indicated mice. Data in A and B are cumulative of multiple experiments, and in C-G are representative of 3 independent experiments, (*n* = 4 per group). In (A, B and F) ^***^p < 0.0001 and in (H) ^***^p = 0.0004 or 0.0006, one-way ANOVA and unpaired *t*-test.

IL-2 signaling in an all-or-none-dependent manner regulates erythropoiesis. This is evident from the severely reduced numbers of Ter119^+^ cells in the BM of *Il2*^−/−^ mice compared to WT and *Il2*^+/-^ mice (Figure 1A). During erythrocyte differentiation in BM, CD71^+^Ter119^-^ erythroid precursor cells differentiate to the CD71^+^Ter119^+^ stage, where upon further signaling they finally develop into CD71^-^Ter119^+^ mature erythrocytes [35]. As reported earlier, lack of IL-2 signaling resulted in a block in erythrocyte differentiation at the transition of CD71^+^Ter119^-^ cells to the CD71^+^Ter119^+^ stage [25]. As a result, in the BM of *Il2*^−/−^ mice an accumulation of CD71^+^Ter119^-^ cells and a paucity of Ter119^+^ cells were observed (Figure 1B). To investigate if NFAT proteins are involved in this defective erythropoiesis in *Il2*^−/−^ mice, we analysed *Nfat* expression in erythrocytes. NFAT involvement in erythropoiesis has been suggested but their exact role is not clear until now [36]. Analysis of BM cells revealed a dose-dependent increase in *Nfatc1*, *Nfatc2* and *Nfatc3* expression in *Il2*^−/−^ mice compared to littermate control mice (Figure 1C). A similar observation in isolated BM Ter119^+^ cells not only revealed erythrocyte-specific *Nfat* expression but also confirmed high *Nfat* expression in *Il2*^−/−^ mice (Figure 1D).

At protein levels, immunofluorescence analysis revealed NFATc1 proteins in WT immature CD71^+^Ter119^-^ cells (Figure 1E, 1F). Again, enhanced levels of NFATc1 in *Il2*^−/−^ CD71^+^Ter119^-^ cells compared to WT cells confirmed an increased NFAT activity in the immature erythrocytes in the absence of IL-2 signaling (Figure 1E, 1F). To further substantiate our observation regarding *Nfat* expression in erythrocytes, we analysed the levels of enhanced green fluorescent protein (eGFP) expression in CD71^+^Ter119^-^ and Ter119^+^ (CD71^+^Ter119^+^ + CD71^-^Ter119^+^) BM cells from *Nfatc1*-*eGfp*-*Bac* transgenic (tg) reporter mice [37]. Detectable GFP levels in both populations in *Nfatc1-eGfp-Bac* tg mice confirmed an erythrocyte-specific *Nfat* expression (Figure 1G, 1H). Again, CD71^+^Ter119^-^ and Ter119^+^ cells from *Il2*^−/−^*Nfatc1-eGfp-Bac* tg mice revealed higher GFP levels compared to *Nfatc1-eGfp-Bac* tg cells, confirming an increased NFAT activity in *Il2*^−/−^ erythrocytes (Figure 1G, 1H). Additionally, GFP analysis also revealed a decrease in *Nfatc1* expression in the more mature Ter119^+^ cells compared to the immature CD71^+^Ter119^-^ cells, both in the *Nfatc1-eGfp-Bac* tg and *Il2*^−/−^*Nfatc1-eGfp-Bac* tg cells (Figure 1G, 1H) suggesting that NFAT activity could influence the transition of CD71^+^Ter119^-^ cells to later stages.

### Enhanced integrin-cAMP signaling in *Il2*^-/-^ erythrocytes

**Figure 2:**
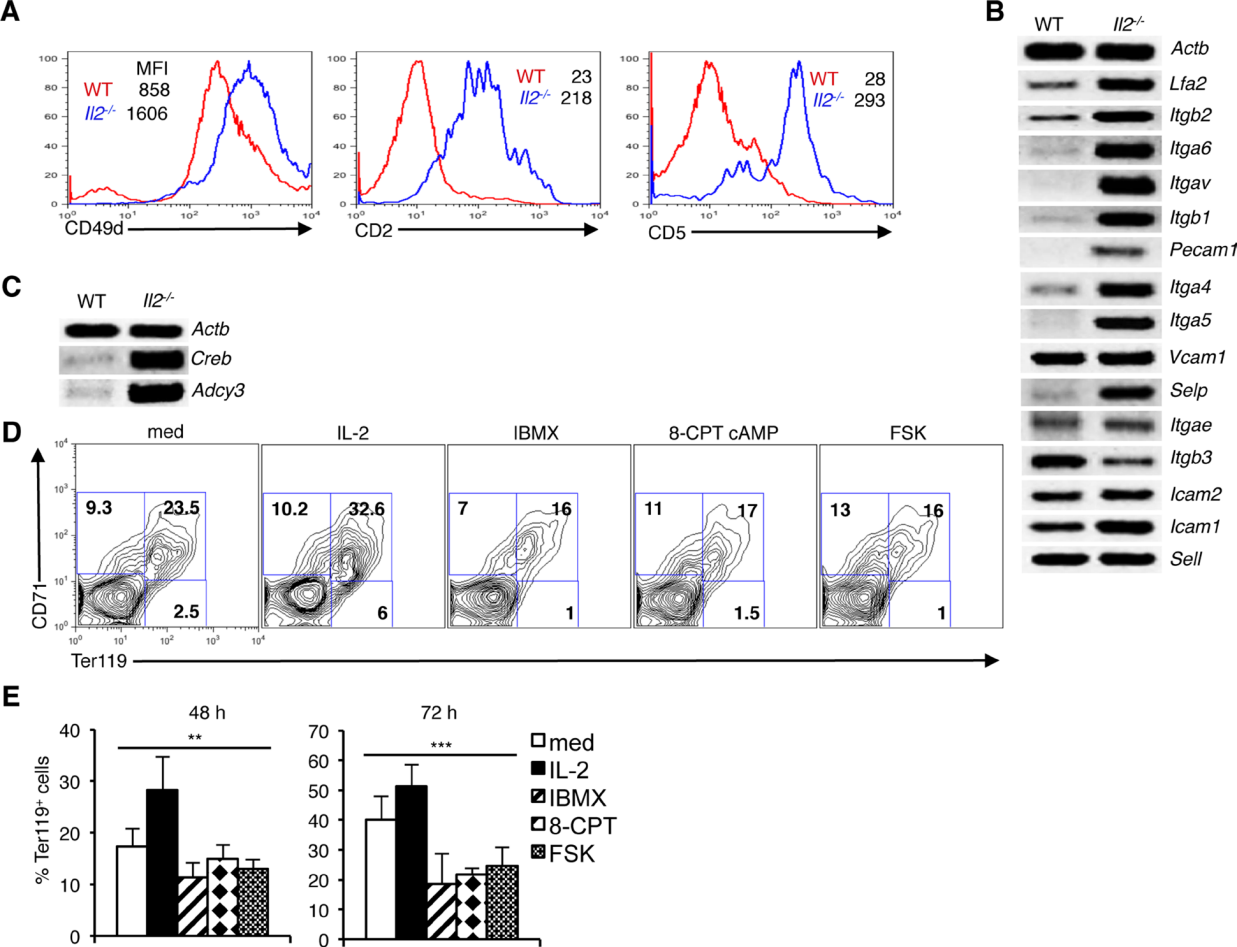
Integrin-cAMP signals influence erythrocyte differentiation (**A**) Expression levels of CD49d, CD2 and CD5 on Ter119^+^ erythroid cells from WT and *Il2*^−/−^ mice. (**B**) Level of mRNA expression of various integrins in *Il2*^−/−^ Ter119^+^ cells compared to WT mice. (**C**) Expression of cAMP signaling-related genes *Adcy3* and *Creb* in *Il2*^−/−^ Ter119^+^ cells compared to that in WT mice. (**D**) Distribution of erythroid cells based on CD71 and Ter119 expression in WT BM cells cultured for 48 h either in medium only or in the presence of IL-2, IBMX, 8-CPT-cAMP or Forskolin (FSK). (**E**) Quantification of Ter119^+^ cells at 48 and 72 h of WT BM cell culture in the absence or presence of IL-2, IBMX, 8-CPT-cAMP or FSK. Data in (A–C) are representative of three and in (D and E) are representative of two independent experiments. Numbers inside the histograms in (A) represent mean fluorescence intensity (MFI) and numbers inside each FACS plot in (D) represent percent respective populations. Data in (E) are presented as mean ± s.d., ^**^p = 0.0019, ^***^p = 0.001, one-way ANOVA.

We have recently reported an integrin-cAMP signaling-mediated regulation of *Nfat* expression in thymocytes and T cells [32]. Involvement of cAMP in integrin signaling has been reported previously [38–40]. Also, a previous study has reported involvement of integrin in erythrocyte development [4]. To investigate whether integrin-cAMP signaling also regulates *Nfat* expression in erythrocytes, we analyzed integrin expression on erythroid cells. Both at protein and mRNA levels, expression of various integrin were detectable in WT BM Ter119^+^ cells (Figure 2A, 2B). Integrin expression was highly variable as some integrin (*Vcam1*, *Itgb3*, *Icam2*, *Icam1* and *Sell*) were expressed at higher level compared to others (*Lfa2*, *Itgb2*, *Itga6*, *Itgav*, *Itgb1*, *Pecam1*, *Itga4*, *Itga5* and *Selp*), (Figure 2B). Interestingly, in *Il2*^−/−^ Ter119^+^ cells a general and strong increase in expression of many integrin was observed (Figure 2A, 2B). Compared to WT cells, *Il2*^−/−^ CD71^+^Ter119^+^ cells showed an increased expression of CD49d, CD2, and CD5 molecules (Figure 2A). Additionally, in *Il2*^−/−^ Ter119^+^ cells an increased expression of other integrin (*Lfa2*, *Itgb2*, *Itga6*, *Itgav*, *Itgb1*, *Pecam1*, *Itga4*, *Itga5* and *Selp*) (Figure 2B) suggested that enhanced integrin-cAMP signals might be involved in the upregulated NFAT expression in these cells. Supporting this conclusion, we observed increased expression of *Adcy3* (adenylate cyclase 3) and *Creb* in *Il2*^−/−^ Ter119^+^ cells compared to WT cells (Figure 2C). Efficient CREB (cAMP-response element binding) binding sequences at *Nfatc1* promoter, as well as Forskolin or cAMP-CREB-mediated *Nfatc1* transcriptional activation has been reported previously [41, 42]. Our observations suggest that an increased integrin-cAMP signaling that operates in *Il2*^−/−^ erythroid cells is the reason behind enhanced NFAT activity in these cells.

To explore whether increased cAMP signaling influences erythrocyte differentiation, we treated *Il2*^−/−^ BM cells with IL-2 or various cAMP-inducing agents. IL-2 treatment enhanced the differentiation of CD71^+^Ter119^+^ cells, whereas increasing cAMP activity by treating cells with IBMX; an inhibitor of phosphodiesterase activity, or 8-CPT-cAMP; a cAMP analog, or with Forskolin reduced this differentiation (Figure 2D, 2E). This suggests that the increased integrin-cAMP signaling in *Il2*^−/−^ mice exerts a negative influence on erythrocyte differentiation.

### Lack of IL-2 signaling impairs *Klf1* expression

**Figure 3:**
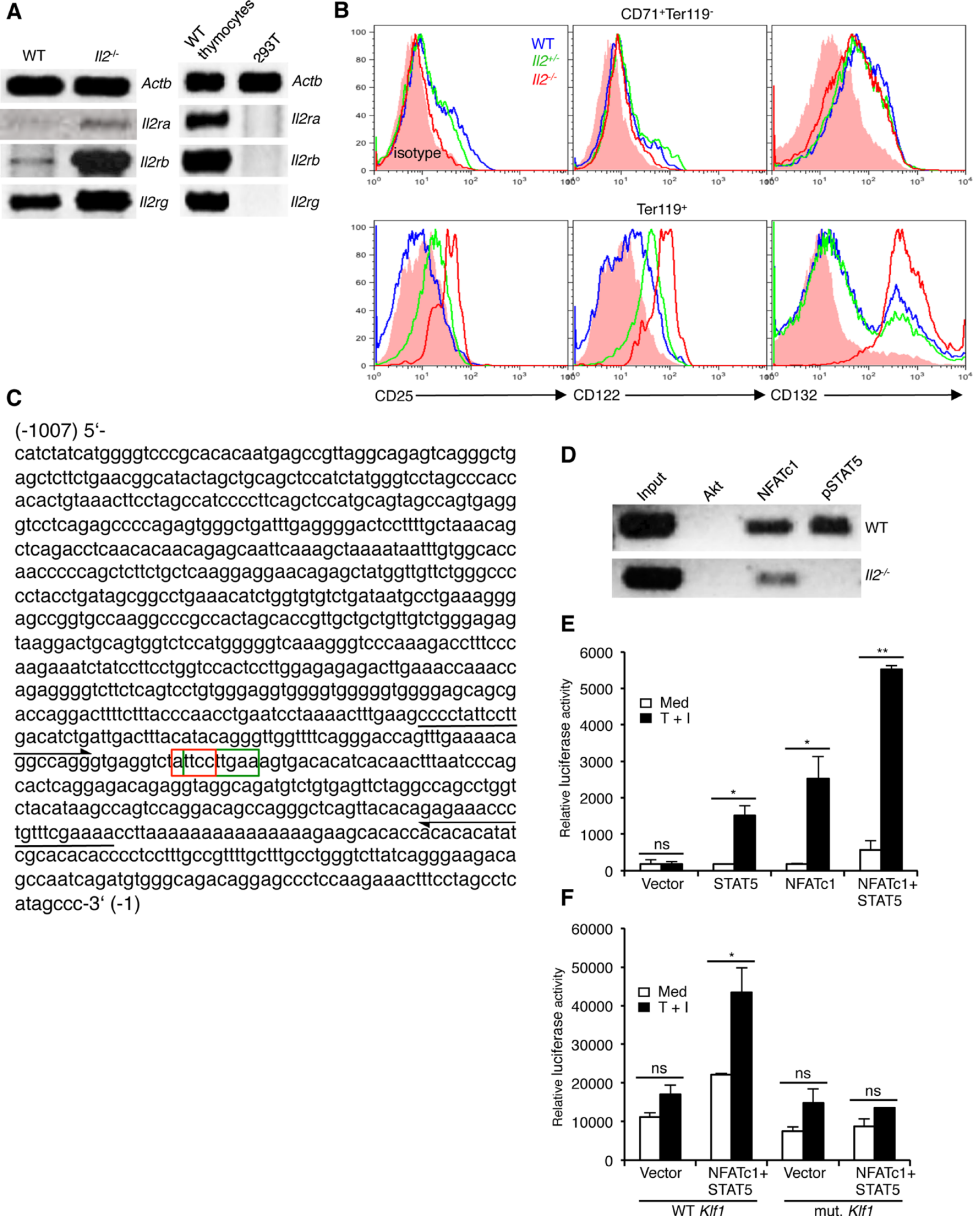
Impaired NFATc1 and STAT5 binding at *Klf1* promoter in *Il2*^−/−^ erythrocytes (**A**) Expression of *Il2ra*, *Il2rb* and *Il2rg* genes in *Il2*^−/−^ Ter119^+^ cells compared to WT cells. WT thymocytes and 293T cells were used as positive and negative controls respectively. (**B**) Intracellular expression of CD25 (IL-2Rα), CD122 (IL-2Rβ) and CD132 (IL-2Rγ) in BM CD71^+^Ter119^-^ and Ter119^+^ (CD71^+^Ter119^+^ + CD71^-^Ter119^+^) cells from WT and *Il2*^−/−^ mice. (**C**) Nucleotide sequence of 1kb DNA element upstream of the transcriptional start site containing the *Klf1* gene promoter. The composite NFATc1 (3′-CCTTA-5′: red boxed) and STAT5 (5′-TTCNNNGAA-3′: green boxed) binding sites are indicated. Arrows in 5′ and 3′ directions indicate the region amplified in ChIP assays for NFATc1 and STAT5 binding to *Klf1* promoter. (**D**) ChIP analysis for NFATc1 and STAT5 binding at the *Klf1* promoter region as indicated in (C) in isolated Ter119^+^ cells from WT and *Il2*^−/−^ mice. ChIP with Akt Abs was used as a negative control. (**E**) Luciferase reporter assay depicting the influence of NFATc1 and STAT5 on *Klf1* promoter activity in unstimulated or TPA + Ionomycin (T + I) stimulated 293 HEK cells. (**F**) Effect of NFATc1 and STAT5 on mutant *Klf1* promoter (TT-GA) activity in unstimulated or TPA + Ionomycin (T + I) stimulated 293 HEK cells. Data in (A, B, E and F) are representative of three, and in (D) are representative of two independent experiments. Data in (E and F) are presented as mean ± s.d., ns = not significant, and in (E) ^*^p = 0.0178 or 0.0333 and ^**^p = 0.0016, and in (F) ^*^p = 0.0415, paired *t*-test.

The enhanced integrin expression in *Il2*^−/−^ Ter119^+^ cells suggest that IL-2 signaling in WT cells most likely keeps the integrin-cAMP signaling in check and, thereby, facilitates differentiation of CD71^+^Ter119^-^ cells to CD71^+^Ter119^+^ stage. If this was the case, immature erythrocytes should respond to IL-2 signals and, therefore, should express the IL-2 receptor (IL-2R). Analysis of IL-2R components revealed undetectable expression of *Il2ra* (*Cd25*), very low level of *Il2rb* (*Cd122*), and a relatively strong expression of *Il2rg* (*Cd132*) in WT erythroid cells (Figure 3A). Interestingly, compared to WT controls, in *Il2*^−/−^ Ter119^+^ cells both at mRNA and protein levels all three IL-2R components were strongly upregulated (Figure 3A, 3B). Intriguingly, WT Ter119^+^ cells expressed higher levels of IL-2Rβ and IL-2Rγ compared to the immature CD71^+^Ter119^-^ cells (Figure 3B). Again, *Il2*^−/−^ Ter119^+^ cells not only expressed much higher levels of IL-2Rβ and IL-2Rγ but also significantly upregulated the expression of IL-2Rα, which was not detectable in WT Ter119^+^ cells (Figure 3B). These observations suggest that in immature erythrocytes IL-2 signals most likely dampen the integrin-cAMP levels to reduce NFAT activity and facilitate their differentiation.

To investigate the functional relevance of enhanced NFAT activity in *Il2*^−/−^ erythroid cells, we hypothesized that most likely it suppresses *Klf1* expression in these cells. Analysis of the DNA element 1kb upstream of the transcriptional start site of *Klf1* gene revealed a composite binding site for NFAT (5′-GGAAA/T-3′) and STAT5 (5′-TTCNNNGAA-3′) (Figure 3C). Chromatin immunoprecipitation analysis revealed a strong NFATc1 binding at the *Klf1* promoter in WT CD71^+^Ter119^-^ cells (Figure 3D). However, we also observed an equally strong binding of STAT5 at the *Klf1* promoter suggesting a possible cooperation between STAT5 and NFATc1 in regulating *Klf1* expression. Accordingly, in reporter assays only STAT5 and NFATc1 together induced strong *Klf1* promoter activity, whereas promoter activity was minimal to STAT5 or NFATc1 activity alone (Figure 3E). Further, STAT5 and NFATc1-induced *Klf1* promoter activity was lost when the composite site was mutated (ATTCCTTGAA - AGACCTTGAA) (Figure 3F). STAT5 activity in response to erythropoietin (Epo) signaling has been reported to critically influence erythrocyte survival and differentiation [23, 43]. Interestingly, despite having increased NFATc1 levels, in *Il2*^−/−^ CD71^+^Ter119^-^ cells a strong impairment in NFATc1 binding to *Klf1* promoter was observed (Figure 3D). This could be due to the absence of STAT5 activity in *Il2*^−/−^ CD71^+^Ter119^-^ cells as they not only are deficient in IL-2 signals, but also lack optimal Epo receptor (EpoR) expression [25]. These observations suggest that IL-2 signaling is essential for optimal *Klf1* expression and thereby promotes erythropoiesis in the BM.

### Enhanced cell death of immature erythrocytes in *Il2*^-/-^ mice

**Figure 4:**
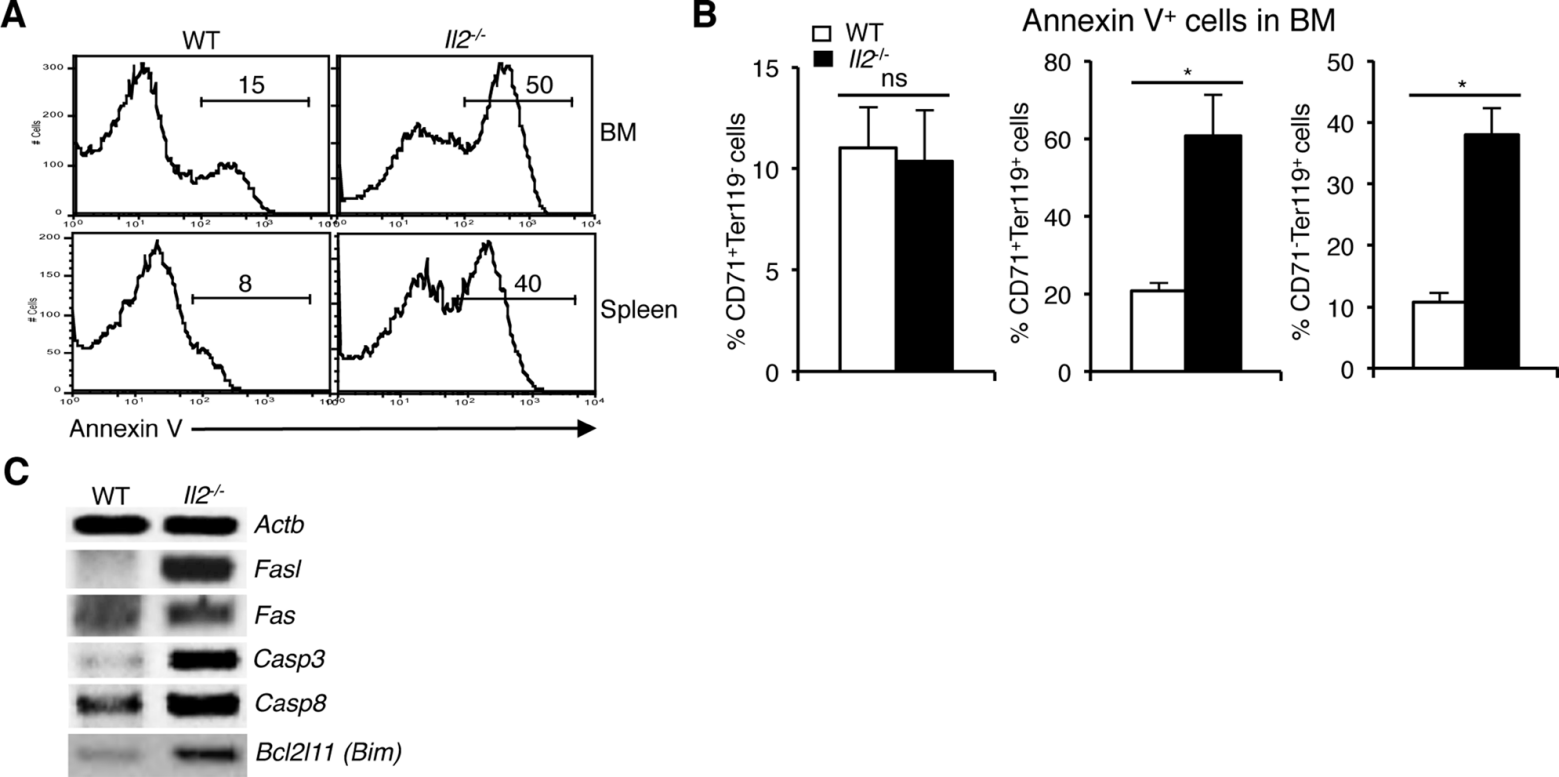
Enhanced NFAT signaling induces apoptosis in *Il2*^−/−^ immature erythrocytes (**A**) Cell death analysis in BM and splenic Ter119^+^ cells from WT and *Il2*^−/−^ mice as revealed by Annexin V staining. (**B**) Quantification of % Annexin V positive cells in BM CD71^+^Ter119^-^, CD71^+^Ter119^+^ and CD71^-^Ter119^+^ fractions from WT and *Il2*^−/−^ mice. (**C**) Expression of apoptosis-related genes in the BM Ter119^+^ cells from *Il2*^−/−^ mice compared to WT controls. Data in (A–C) are representative of three independent experiments. Number inside each histogram represents percent Annexin V positive population. Data in (B) are presented as mean ± s.d., ns = not significant and ^*^p = 0.7376 or 0.0141, paired *t*-test.

To investigate whether enhanced NFAT activity exerts a negative influence on their survival, we analysed cell death in *Il2*^−/−^ erythroid precursor cells. Annexin V analysis revealed a strong increase in apoptosis (50% vs 15% in BM and 40% vs 8% in spleen) of *Il2*^−/−^ erythroid cells compared to WT mice (Figure 4A). The increased cell death in *Il2*^−/−^ mice was mostly in the CD71^+^Ter119^+^ and CD71^-^Ter119^+^ compartments as cell death in the CD71^+^Ter119^-^ stage was similar to that in WT mouse (Figure 4B). These observations suggest that anemia in *Il2*^−/−^ mice is a combinatorial effect of *Klf1* dysregulation and subsequent apoptosis of immature erythrocytes. Agreeing with this, we observed an increase in expression of *Fasl*, a prominent target of NFAT [44, 45], and also of *Fas*, *Casp3*, *Casp8* and *Bim* in *Il2*^−/−^ erythroid cells (Figure 4C). NFAT proteins have been shown to regulate activation-induced cell death (AICD) of T cells following an immune response in a Fas-FasL-dependent manner [46, 47]. Thus, enhanced Fas-FasL activity as well as upregulated activity of other apoptosis-promoting molecules in IL-2-deficient erythroid cells results in increased cell death leading to anemia development in *Il2*^−/−^ mice.

### Ablation of NFAT activity promotes erythropoiesis

**Figure 5:**
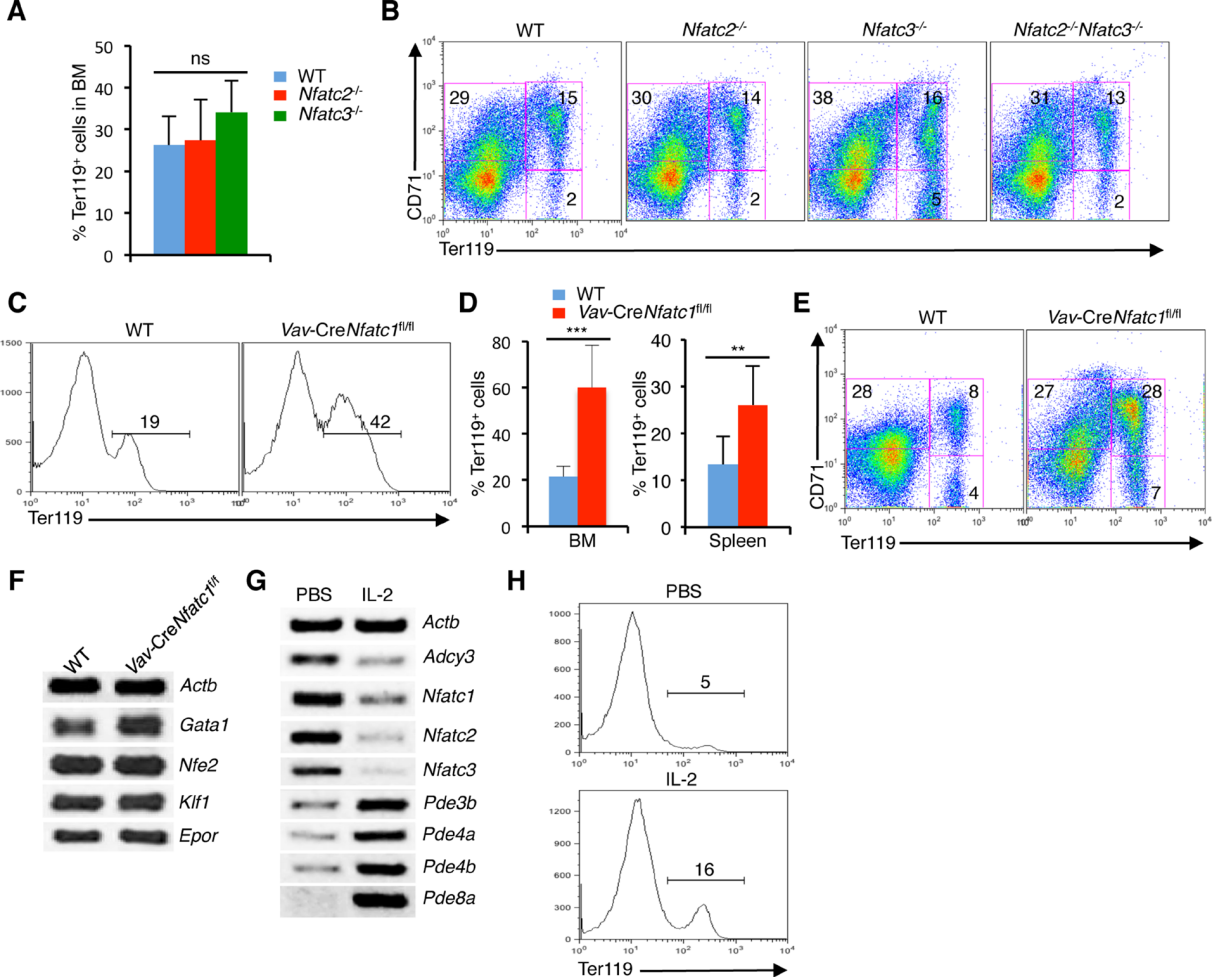
Reduction in NFATc1 activity facilitates erythropoiesis (**A**) Quantification of BM Ter119^+^ cells in *Nfatc2*^−/−^ and *Nfatc3*^−/−^ mice compared to littermate control mice. (**B**) Distribution of erythroid cells based on CD71 and Ter119 expression in the BM of *Nfatc2*^−/−^, *Nfatc3*^−/−^ and *Nfatc2*^−/−^*Nfatc3*^−/−^ mice compared to WT mice. (**C**) Ter119^+^ cells distribution in the BM of *Vav*-Cre*Nfatc1*^fl/fl^ mice compared to littermate control mice. (**D**) Quantification of percent Ter119^+^ cells in BM and spleen of *Vav*-Cre*Nfatc1*^fl/fl^ mice compared to littermate WT mice. (**E**) Erythrocyte differentiation profile based on CD71 and Ter119 expression in the spleen of *Vav*-Cre*Nfatc1*^fl/fl^ mice compared to WT littermates. (**F**) Expression of *Gata1*, *Nfe2*, *Klf1* and *Epor* genes in BM Ter119^+^ cells from WT and *Vav*-Cre*Nfatc1*^fl/fl^ mice. (**G**) *Nfat* and cAMP signaling-related genes expression in BM Ter119^+^ cells from PBS or IL-2 treated *Il2*^−/−^ mice. (**H**) Distribution of Ter119^+^ cells in the BM of PBS or IL-2 treated *Il2*^−/−^ mice. Data in (A–H) are representative of three or more independent experiments. Numbers inside each FACS plot represent percent respective populations. Data in (A and D) are presented as mean ± s.d., ns = not significant, in (D) ^**^p = 0.0063 and ^***^p < 0.0001, one-way ANOVA and paired *t*-test.

To investigate which NFAT protein is responsible for the defective erythropoiesis in *Il2*^−/−^ mice, we analyzed various *Nfat* mutant mice. Analysis of *Nfatc2*^−/−^ or *Nfatc3*^−/−^ mice revealed comparable numbers of Ter119^+^ cells in BM to that of littermate WT mice (Figure 5A). Further analysis for differentiation stages showed neither *Nfatc2*^−/−^ nor *Nfatc3*^−/−^ mice had any defect at the CD71^+^Ter119^+^ stage, and they differentiated to CD71^-^Ter119^+^ erythrocytes similar to that in control mice (Figure 5B). Even the combined loss of NFATc2 and NFATc3 activity (*Nfatc2*^−/−^*Nfatc3*^−/−^ mice) did not impair erythropoiesis in the BM (Figure 5B). These observations suggest that NFATc1 is the key erythrocyte-specific NFAT protein and overactivity of NFATc1 is probably inducing the erythropoietic defects and anemia in *Il2*^−/−^ mice.

*Nfatc1*^−/−^ mice are embryonic lethal [48]. Therefore, we analyzed *Vav*-Cre*Nfatc1*^fl/fl^ mice, which revealed an enhanced erythropoiesis, as they had significantly increased Ter119^+^ population both in BM and spleen compared to littermate WT mice (Figure 5C, 5D). This was in contrast with the normal erythropoiesis observed in *Nfatc2*^−/−^, *Nfatc3*^−/−^ and *Nfatc2*^−/−^*Nfatc3*^−/−^ mice (Figure 5A, 5B) indicating that manipulation of NFATc1 activity can influence erythropoiesis. Also, the increased Ter119^+^ population in *Vav*-Cre*Nfatc1*^fl/fl^ mice was in contrast to the severely reduced numbers of Ter119^+^ cells in *Il2*^−/−^ mice, suggesting that enhanced NFATc1 activity most likely has a negative influence on BM erythropoiesis. In line with the increase in Ter119^+^ cells, differentiation of CD71^+^Ter119^-^ cells to Ter119^+^ stages was enhanced in *Vav*-Cre*Nfatc1*^fl/fl^ mice compared to WT controls (Figure 5E). Further, *Vav*-Cre*Nfatc1*^fl/fl^ Ter119^+^ cells expressed normal levels of *Klf1* and *Epor*, which were suppressed in the *Il2*^−/−^ mice (Figure 5F).

Next, we analysed if IL-2-deficiency is leading to enhanced NFAT activity, treatment with IL-2 should reverse this in *Il2*^−/−^ mice. Interestingly, IL-2 treatment effectively downregulated *Nfat* expression in *Il2*^−/−^ Ter119^+^ cells (Figure 5G). Simultaneously, a strong downregulation in *Adcy3*, and upregulation in several phosphodiesterases (*Pde3b*, *Pde4a*, *Pde4b*, and *Pde8a*) in *Il2*^−/−^ Ter119^+^ cells was observed. These observations further emphasize that the increased cAMP signaling was responsible for the enhanced NFAT activity in *Il2*^−/−^ mice. As a result of downregulation in NFAT activity, erythropoiesis in IL-2 treated *Il2*^−/−^ mice returned to normal, as more Ter119^+^ cells appeared in IL-2 treated mice compared to PBS treated controls (Figure 5H). Thus, by manipulating cAMP levels, IL-2 signals regulate NFAT activity in erythroid cells.

## DISCUSSION

Our observation about *Nfat* expression in erythrocytes suggests they might have a role to play in erythropoiesis, and the increased NFAT activity in *Il2*^−/−^ erythroid cells (Figure 1D–1H) could be one of the reasons that the *Il2*^−/−^ mice suffer from severe anemia. We have shown previously that IL-2 critically regulates BM erythropoiesis in a T_reg_-dependent manner, and restoration of T_reg_ activity or abolition of IFN-γ activity significantly reversed anemia in *Il2*^−/−^ mice [25]. Also, recently we have reported that in a T_reg_-dependent manner IL-2 signaling plays an indispensable role in the maintenance of hematopoietic stem cell (HSC) integrity [49]. However, the lack of T_reg_ activity though severely impairs BM erythropoiesis, additional factors and signaling molecules might be involved in the drastically reduced number of RBCs in *Il2*^−/−^ mice. One possible reason for anemia in *Il2*^−/−^mice could be increased cell death of the differentiating erythrocytes. Our observation regarding increased apoptosis of Ter119^+^ populations in *Il2*^−/−^ mice supports this notion (Figure 4A, 4B).

Most likely NFAT activity is essential at the early stages in the CD71^+^Ter119^-^ cells and needs to be downregulated as the erythrocytes mature. This is evident from our analysis of the *Nfatc1-eGfp-Bac* tg reporter mice, where GFP levels were highest in the CD71^+^Ter119^-^ cells but was reduced in the Ter119^+^ cells (Figure 1G, 1H). This probably happens via IL-2 signaling-mediated downregulation in cAMP levels in these cells. Our observations of downregulated *Adcy3* and upregulated phosphodiesterases expression in IL-2 treated *Il2*^−/−^ mice support this hypothesis (Figure 5G). Under this circumstance, *Nfat* expression was suppressed and erythropoiesis in *Il2*^−/−^ mice was restored (Figure 5G, 5H). We have shown previously that integrin-cAMP signaling induces *Nfat* expression in thymocytes and T cells [32]. Similar to thymocytes, erythrocytes also express various integrin and their levels were strongly upregulated in the IL-2-deficient Ter119^+^ cells (Figure 2A, 2B). Increasing intracellular cAMP levels clearly has a negative influence on erythrocyte differentiation as fewer Ter119^+^ cells developed in *in vitro* BM cultures where cAMP levels were enhanced (IBMX, 8-CPT-cAMP or FSK treated cells) compared to the IL-2-treated cells (Figure 2D, 2E). This suggests that the developing erythrocytes are sensitive to IL-2 signaling.

IL-2 receptor expression in erythrocytes is so far not known. However, analysis of ‘ErythronDB’, the database containing genes expressed during erythropoiesis, shows robust expression of *Il2rg* in all stages of erythrocyte development [50, 51]. The absence of IL-2Rα and low levels of IL-2Rβ and IL-2Rγ expression in WT Ter119^+^ cells (Figure 3A, 3B) suggest that in erythrocytes IL-2 signals most likely are transduced via the intermediate-affinity IL-2Rβ + IL-2Rγ receptors, similar to that in naïve T cells [18]. However, the upregulated expression of IL-2R components in *Il2*^−/−^ erythrocytes (Figure 3A, 3B) is quite surprising, and we still do not know the mechanism behind it. The presence of the composite NFAT and STAT5 binding sequence at the *Klf1* promoter suggests that optimal NFAT and STAT5 activity might be an essential factor that regulates *Klf1* expression in erythrocytes (Figure 3C). Enhanced NFAT activity in absence of STAT5 activity in *Il2*^−/−^ erythrocytes failed to induce optimal *Klf1* expression resulting in a differentiation block at the CD71^+^Ter119^-^ to CD71^+^Ter119^+^ transition. Further, the induction of *Klf1* promoter activity in response to combined NFATc1 and STAT5 activity (Figure 3E), and the loss of promoter activity when the composite site was mutated (Figure 3F) suggests that an optimal activity of essential TFs during erythrocyte differentiation stages is critical for normal erythropoiesis.

Our analysis of various *Nfat* mutant mice suggests that NFATc1 activity most likely plays an important role in erythrocyte differentiation. Neither single nor combined loss of NFATc2 and NFATc3 activity had any influence on Ter119^+^ cell development in the BM (Figure 5A, 5B). However, loss of hematopoietic cells-specific NFATc1 activity clearly induced augmented differentiation of CD71^+^Ter119^-^ cells to the CD71^+^Ter119^+^ stage, which resulted in the generation of more Ter119^+^ erythrocytes in *Vav*-Cre*Nfatc1*^fl/fl^ mice compared to WT controls (Figure 5C–5E). This was similar to what has been reported for mice deficient in Desert Hedgehog (*Dhh*) signalling [52]. The significant improvement in erythropoiesis in the IL-2 treated *Il2*^−/−^ mice (Figure 5H), where upon IL-2 signaling *Nfat* expression was strongly reduced (Figure 5G) also supports the point that enhanced NFAT activity is harmful for erythrocyte differentiation.

**Figure 6:**
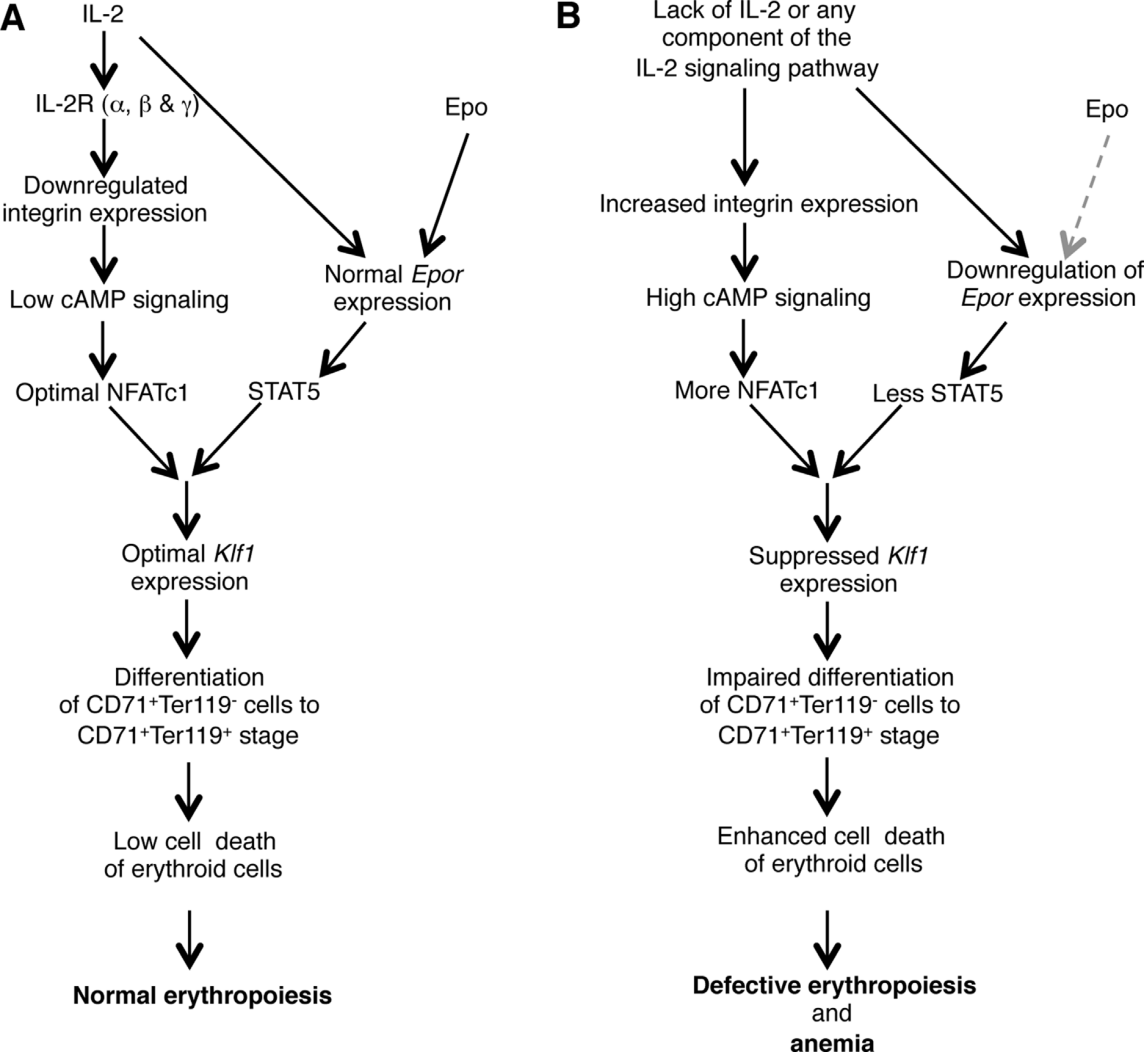
Model showing the role of IL-2 in maintaining BM erythropoiesis (**A**) IL-2 signals are essential to lower the integrin-cAMP-NFAT signaling, and in facilitating optimal KLF1 and EpoR expression, which results in normal erythropoiesis in the BM. (**B**) Deficiency in IL-2 or of any component of IL-2 signaling pathway leads to upregulated integrin-cAMP-NFAT signaling as well as suppressed *Klf1* and *Epor* expression. Both these defects combinedly result in enhanced cell death of immature erythrocytes leading to the development of severe anemia in IL-2 signaling-deficient mice. Grey dashed arrow represents lack of Epo signaling.

Altogether, our observations suggest a critical role for IL-2 in maintaining steady state RBC number by regulating the integrin-cAMP-NFAT signaling axis (Figure 6). Manipulation of this signaling pathway could open up new avenues to modulate erythropoiesis and to prevent anemia development.

## MATERIALS AND METHODS

### Mice

C57BL/6 wild-type, *Il2*^−/−^, *Nfatc1-eGfp-Bac* tg, *Il2*^−/−^*Nfatc1-eGfp-Bac* tg, *Nfatc1*^fl/fl^, *Vav*-Cre*Nfatc1*^fl/fl^, *Nfatc2*^−/−^, *Nfatc3*^−/−^, and *Nfatc2*^−/−^*Nfatc3*^−/−^ mice, all on C57BL/6 background and of 3–8 weeks age unless mentioned otherwise were used throughout the study. Animals were housed in the central animal facility (ZEMM) of the University of Würzburg, according to standard animal care protocols. All animal experiments were performed taking utmost care, and were according to established guidelines (approved by the Regierung von Unterfranken, Wuerzburg, Permit Number 55.2-2531.01-53/10B).

### Flow cytometry

For flow cytometry, and Ter119^+^ BM cell isolation all antibodies were purchased either from BD Pharmingen or eBioscience. Anti-CD2 (RM2-5), anti-CD5 (53-7.3), anti-CD25 (PC61), anti-CD49d (R1-2), anti-CD71 (C2), anti-CD122 (TM-b1), anti-CD132 (TUGm2), anti-Ter119 (TER-119), annexin V and isotype-matched control antibodies either directly conjugated with fluorochromes or with biotin were used throughout this study. Biotinylated antibodies were revealed with secondary streptavidin-allophycocyanin or phycoerythrin-Cy5 (PE-Cy5) antibodies. Flow cytometry and data analysis were performed following standard procedure using FACSCalibur and FlowJo software.

### Cell isolation and sorting

For cell isolation and sorting, BM cells from both hind limbs were collected and single cell suspension was prepared. Ter119^+^ BM cells were isolated using anti-Ter119 microbeads (Miltenyi Biotec) following manufacturer’s protocol. For cell sorting, BM cells were incubated with anti-CD71 and anti-Ter119 antibodies. CD71^+^Ter119^-^, CD71^+^Ter119^+^ and CD71^-^Ter119^+^ erythroid cells were sorted by using a FACSAria (BD Biosciences) flow cytometer.

### Immunofluorescence staining

Sorted CD71^+^Ter119^-^ BM cells from WT or *Il2*^−/−^ mice were immunostained with NFATc1 (Santa Cruz, sc14034) antibodies following previously published protocol [30]. DAPI was used to confirm nuclear staining.

### Intracellular staining

For intracellular CD25 (IL-2Rα), CD122 (IL-2Rβ) and CD132 (IL-2Rγ) staining, 5 × 10^6^ freshly prepared BM cells from WT and *Il2*^−/−^ mice were used. Cells were first surface stained for CD71 and Ter119 followed by intracellular CD25, CD122 and CD132 staining according to eBioscience Foxp3 staining protocol. IL-2 receptors expression in erythroid cells was analyzed by gating on CD71^+^Ter119^-^, CD71^+^Ter119^+^ and CD71^-^Ter119^+^ erythroid cells.

### Cell death analysis

1 × 10^6^ freshly isolated WT or *Il2*^−/−^ BM and spleen cells were used to evaluate apoptosis of total Ter119^+^ cells, or of gated CD71^+^Ter119^-^, CD71^+^Ter119^+^ and CD71^-^Ter119^+^ erythroid cells. BM cells were stained for CD71 and Ter119 and subsequently, live or dead cell discrimination was performed with annexin V staining following manufacturer’s protocol (BD Biosciences).

### Chromatin immunoprecipitation

5–8 × 10^6^ WT or *Il2*^−/−^ BM CD71^+^Ter119^+^ cells were used for each ChIP assay following the Miltenyi Biotec ChIP protocol. 8–10 µg of NFATc1 (Santa Cruz; sc-7294), pSTAT5 (NEB; 9314), and Akt (NEB; 9272) antibodies were used for immunoprecipitation. DNA fragments were purified and used to amplify the *Klf1* promoter region bound to NFATc1 or STAT5. Primers used to amplify the *Klf1* promoter region are mentioned in the Supplementary Table 1.

### *In vivo* injections

6 weeks old male and female *Il2*-/- mice were injected on every alternate day with 1 µg recombinant murine IL-2 (rmIL-2, Peprotech) or an equal volume of PBS intraperitoneally for two consecutive weeks. Four days after getting the last injection, mice were analyzed to study the effects of IL-2 on erythropoiesis in the BM.

### *In vitro* culture assay

3 × 10^6^ freshly isolated WT BM cells were either cultured in RPMI-1640 medium supplemented with 10% FCS only, or in presence of IL-2 (1 µg), IBMX (0.5 mM), 8-CPT-cAMP (50 µM) or Forskolin (50 µM). 48 or 72 h later cells were analyzed to investigate the influence of IL-2 or various cAMP-inducing agents on erythrocyte differentiation.

### Semiquantitative RT-PCR

Total BM cells or magnetically isolated Ter119^+^ cells from WT or *Il2*^−/−^ mice were used to synthesize cDNA using Miltenyi Biotec cDNA synthesis kit and protocol. Semiquantitative RT-PCR was performed to investigate the expression of indicated genes. Primer sequences are available in the supplementary information online.

### Luciferase reporter assays

The 1007bp *Klf1* promoter fragment was generated by amplification from murine genomic DNA (chr8: 87424731-87426061) using following primers: Fw.5´-AACTCGAGCATCTATCATGGGGTCCCGC-3´ and Rev.5´-TTAAGCTTGGGCTATGAGGCTAGGAAAG-3´. The blunt ended left (XhoI) and right (HindIII) arms were cloned into the corresponding XhoI and HindIII sites of pGL3 basic vector. 50 ng of murine *Klf1* luciferase reporter construct containing the 1007bp DNA fragment spanning the promoter region (-960 to +47 bp) was co-transfected along with 500 ng control vectors, or expression vectors for a constitutively active STAT5 (STAT5ca) or NFATc1 (Nc1) alone, or with both STAT5 and NFATc1 into 293 HEK cells by polyethylenimine (PEI) (St. Louis, Missouri, USA) transfection method. 36 h post-transfection cells were left unstimulated or stimulated with PMA plus Ionomycin (100 ng/ml each, Calbiochem) for 12 h. Afterwards, luciferase activity representing the *Klf1* promoter transactivation was measured using a MicroLumat LB 96P (EG&G Berthold) luminometer. For mutant *Klf1* promoter construct, mutations in the composite STAT5-NFAT binding site (TT-GA) were introduced by site directed mutagenesis and promoter activity was assessed in response to STAT5 and NFATc1 activity.

### Statistics

Data are presented as mean ± s.d. Statistical significance was assessed using Student’s *t*-test for comparison between two groups and ANOVA for differences between groups.

## SUPPLEMENTARY MATERIALS TABLE




