# Supplementary material for: NFAT-mediated defects in erythropoiesis cause anemia in Il2−/− mice
Source: Oncotarget. 2017 Dec 28;9(11):9632–44. doi: 10.18632/oncotarget.23745 (PMC5839390; doi:10.18632/oncotarget.23745)
Supplement: Supplementary file 1 [file oncotarget-09-9632-s001.pdf]

NFAT-mediated defects in erythropoiesis cause anemia in *Il2*$^{-/-}$ mice

SUPPLEMENTARY MATERIALS

Supplementary Table 1: List of RT-PCR and ChIP primers. See_Supplementary_Table 1
