# Supplementary material for: NFAT-mediated defects in erythropoiesis cause anemia in Il2−/− mice
Source: Oncotarget. 2017 Dec 28;9(11):9632–44. doi: 10.18632/oncotarget.23745 (PMC5839390; doi:10.18632/oncotarget.23745)
Supplement: Supplementary file 2 [file oncotarget-09-9632-s002.docx]

**Supplementary Table 1: LIST OF RT-PCR and ChIP PRIMERS**

| **Gene** | **Primer Sequence** | **Product Size** |
| --- | --- | --- |
| *Actb* | For: 5’-CCAGGTCATCACTATTGGCAAGGA-3’  Rev: 5’-GAGCAGTAATCTCCTTCTGCATCC-3’ | 223 bp |
| *Adcy3* | For: 5’- AGATGGAAACACGCTACTCG -3’  Rev: 5’- AACATTGGCCATAACCAGAA -3’ | 309 bp |
| *Bcl2l11 (Bim)* | For: 5’-TACCAGATCCCCACTTTTCA-3’  Rev: 5’-CATTTGAGGGTGGTCTTCAG-3’ | 331 bp |
| *Casp3* | For: 5’-AGCAAGTCAGTGGACTCTGG-3’  Rev: 5’-AAGAGTTTCGGCTTTCCAGT-3’ | 408 bp |
| *Casp8* | For: 5’-AGAACAAACCTCGGGGATAC-3’  Rev: 5’-AGGCACTCCTTTCTGGAAGT-3’ | 427 bp |
| *Creb* | For: 5’- TGCCACATTAGCCCAGGTAT -3’  Rev: 5’- GTACCCCATCCGTACCATTG -3’ | 506 bp |
| *Epor* | For: 5’- GGACACCTACTTGGTATTGG -3’  Rev: 5’- GACGTTGTAGGCTGGAGTCC -3’ | 452 bp |
| *Fas* | For: 5’-GAGGACTGCAAAATGAATGG-3’  Rev: 5’-GGGTCATCCTGTCTCCTTTT-3’ | 411 bp |
| *Fasl* | For: 5’-CACCTGTGTCACCACTACCA-3’  Rev: 5’-GCGGTTCCATATGTGTCTTC-3’ | 352 bp |
| *Gata1* | For: 5’-ATTCCTGGGGGCTCACCTTATG-3’  Rev: 5’-TCCACAGTTCACACACTCTCTGGC-3’ | 366 bp |
| *Icam1* | For: 5’- CCAAGAAACGCTGACTTCAT -3’  Rev: 5’- CGACCCTTATGAGAAAAGCA -3’ | 327 bp |
| *Icam2* | For: 5’- GAAGCCACAGAGTCTTGGAA -3’  Rev: 5’- TCAGTGTGACTTGAGCTGGA -3’ | 244 bp |
| *Il2ra* | For: 5’- CACAACAGACATGCAGAAGC -3’  Rev: 5’- TTCTGCTCTTCCTCCATCTG -3’ | 494 bp |
| *Il2rb* | For: 5’- AGCCTGTCCCTCTACGTCTT -3’  Rev: 5’- GGGCCTCAAATTCCAAGTAT -3’ | 487 bp |
| *Il2rg* | For: 5’-CCAAGGTCCTCATGTCCAGT-3’  Rev: 5’-CCTGGAGCTGGACAACAAAT-3’ | 329 bp |
| *Itgb1* | For: 5’- AAGACATGGACGCTTACTGC -3’  Rev: 5’- ATGGACCAGTGTCCAAAGAA -3’ | 267 bp |
| *Itgb2* | For: 5’- TAATGCAAGTTGCTGCATGT -3’  Rev: 5’- GCTGGAGTCGTCAGACAGTT -3’ | 332 bp |
| *Itgb3* | For: 5’- ATACCAGGGAGGACCTTCAG -3’  Rev: 5’- TCCTTCCCTGCTAGTTTCCT -3’ | 257 bp |
| *Itga4* | For: 5’- AAGCCAGCGTTCATATTCAG -3’  Rev: 5’- ATCCAGCCTTCCACATAACA -3’ | 277 bp |
| *Itga5* | For: 5’- GTACCTGGGTGACAAGAACG -3’  Rev: 5’- GTTCAGGTTCTTGCTGAGGA -3’ | 323 bp |
| *Itga6* | For: 5’- TGAGGTGTGTGAACATCAGG -3’  Rev: 5’- TAGAGCCAGCATCAGAATCC -3’ | 306 bp |
| *Itgae* | For: 5’- AATGGCATTCAGTGGTCTGT -3’  Rev: 5’- TCCTTGTGCTCTCCAAGTTC -3’ | 346 bp |
| *Itgav* | For: 5’- GGAGAACCAGAACCATTCCT -3’  Rev: 5’- TTGCTCTTCTTGAGGTGGTC -3’ | 272 bp |
| *Klf1* | For: 5’-GATCGCCGGAGACGCAGGCT-3’  Rev: 5’-TCCCCAGTCCTTGTGCAGGA-3’ | 363 bp |
| *Lfa2* | For: 5’-GCACAAATGGGATGACTAGG-3’  Rev: 5’-CCCCCACTGTGACATAGAAG-3’ | 322 bp |
| *Nfatc1* | For: 5’- GACTTCGATTTCCTCTTCGAGTTC -3’  Rev: 5’- CTCGATTCTCGGACTCTCCAG -3’ | 297 bp |
| *Nfatc2* | For: 5’-GGGTTCGGTGAGTGACAGTT-3’  Rev: 5’-CTCCTTGGCTGTTTGGGATA-3’ | 371 bp |
| *Nfatc3* | For: 5’-CCGATGACTACTGCAAACTGTGG-3’  Rev: 5’-TTTGAATACTTGGGCACTCAAAGG-3’ | 343 bp |
| *Nfe2* | For: 5’-GAGCCCTGGCCATGAAGATTCC-3’  Rev: 5’-CACCATCAGCAGCCTGTTGCAG-3’ | 391 bp |
| *Pde3b* | For: 5’- CAGTAGCTTGATGGGTGCTT -3’  Rev: 5’- AGACGATGACCTCTGCTTTG -3’ | 341 bp |
| *Pde4a* | For: 5’-CACAGCCTCTGTGGAGAAGT-3’  Rev: 5’-GGAGAGTTTCCTCAGTGCAA-3’ | 305 bp |
| *Pde4b* | For: 5’-CCAGCTAGAGACCATCCAGA-3’  Rev: 5’-GAAGATGTTAAGGCCCCATT-3’ | 367 bp |
| *Pde7b* | For: 5’-GTTTGAGAGCCCTGAACAGA-3’  Rev: 5’-CCAGACTGTTCCCATTTGTC-3’ | 358 bp |
| *Pde8a* | For: 5’-AGCTGGGTTTAAGTGCACAG-3’  Rev: 5’-TACAAGCCCTGAGTTTCAGC-3’ | 338 bp |
| *Pecam1* | For: 5’- TTGGCACAACAAACAAGCTA -3’  Rev: 5’- GAAATCTTCTCGCTGTTGGA -3’ | 292 bp |
| *Sell* | For: 5’- CGCTCATTCATCCCATTAAC -3’  Rev: 5’- GCAAGGAGTCTGAGTTTCCA -3’ | 224 bp |
| *Selp* | For: 5’-TCTTGGGAATTCCACCTACA-3’  Rev: 5’-GGGCAGGAAGTGATGTTATG-3’ | 339 bp |
| *Vcam1* | For: 5’- CTGTACATCCCTCCACAAGG -3’  Rev: 5’- ACACGTCAGAACAACCGAAT -3’ | 321 bp |
|  | **ChIP Primers** |  |
| *Klf1* | For: 5’-CCCCTATTCCTTGACATCTG-3’  Rev: 5’-TTTTCGAAACAGGGTTTCTC-3’ | 233 bp |
